# Reliability of Growth Indicators and Efficiency of Functional Treatment for Skeletal Class II Malocclusion: Current Evidence and Controversies

**DOI:** 10.1155/2017/1367691

**Published:** 2017-01-11

**Authors:** Giuseppe Perinetti, Luca Contardo

**Affiliations:** Department of Medical, Surgical and Health Sciences, School of Dentistry, University of Trieste, Trieste, Italy

## Abstract

Current evidence on the reliability of growth indicators in the identification of the pubertal growth spurt and efficiency of functional treatment for skeletal Class II malocclusion, the timing of which relies on such indicators, is highly controversial. Regarding growth indicators, the hand and wrist (including the sole middle phalanx of the third finger) maturation method and the standing height recording appear to be most reliable. Other methods are subjected to controversies or were showed to be unreliable. Main sources of controversies include use of single stages instead of ossification events and diagnostic reliability conjecturally based on correlation analyses. Regarding evidence on the efficiency of functional treatment, when treated during the pubertal growth spurt, more favorable response is seen in skeletal Class II patients even though large individual responsiveness remains. Main sources of controversies include design of clinical trials, definition of Class II malocclusion, and lack of inclusion of skeletal maturity among the prognostic factors. While no growth indicator may be considered to have a full diagnostic reliability in the identification of the pubertal growth spurt, their use may still be recommended for increasing efficiency of functional treatment for skeletal Class II malocclusion.

## 1. Background

It has been reported decades ago that the growth rate of the mandible is not constant throughout development [1–3] showing a peak during puberty [1, 2, 4, 5]. However, the intensity, onset, and duration of the pubertal growth peak (including mandibular growth peak) are subjected to noteworthy individual variations [1, 3–5]. A deficient mandibular growth on the sagittal plane is the most frequent diagnostic finding in skeletal (and dental) Class II malocclusion that occurs in up to one-third of the population [6, 7]. Thus, a therapy able to enhance mandibular growth is indicated in skeletal Class II patients [8]. In this regard, animal studies have shown that forward mandibular displacement enhances condylar growth resulting in significant mandible elongation [9, 10]. Consequently, a wide range of functional appliances (either removable or fixed) have been developed to stimulate mandibular growth by forward posturing of the mandible.

To date, the efficiency of functional treatment for skeletal Class II malocclusion is still controversial with reviews reporting very limited [11–13], partial [14–16], or relevant [17–19] effects of such treatment in terms of induced mandibular growth. Among the reasons for such inconsistencies is the timing, that is, skeletal maturity [18, 20, 21], during which treatment is performed. Clinical trials indicated that the functional treatment for skeletal Class II malocclusion is efficient when performed during the pubertal growth spurt [22–26] and without clinically relevant effects when performed before [27–29].

Therefore, over the last six decades, efforts have been carried out to find reliable and reproducible indicators of skeletal maturity in individual subjects [5, 20, 30–33]. These indicators have included radiographic hand and wrist maturational (HWM) methods [30, 34, 35], third finger middle phalanx (MPM) method [36–38], cervical vertebral maturational (CVM) methods [20, 33, 39], dental maturation [31, 32, 40] and dental emergence [32, 41], chronological age [5, 41], and noninvasive biomarkers from serum [42, 43] or gingival crevicular fluid (GCF) [44, 45].

## 2. Common Issues related to the Investigation and Use of the Skeletal Maturity Indicators

Current evidence on the reliability of the different growth indicators and consequent definition of treatment timing is highly controversial. Contrasting results have been reported on the capability of the growth indicators (mainly the CVM method) in the identification of the mandibular growth peak [46–53] and on the efficiency of functional treatment for Class II malocclusion [13, 18]. The investigation on growth indicators has common sources of controversies for all the indicators and specific issues related to each indicator. Herein, common controversial issues to all indicators are listed, while specific issues and controversies on the functional treatment are reported below.

### 2.1. Stages versus Ossification Events

In using radiographical indicators of growth phase that are based on sequential discrete stages, an important distinction has to be made between stages and ossification events [54, 55]. The stages are specific periods in the development of a bone that have been described in that particular rating method, while an ossification event occurs when a given stage matures into the following one [54, 55]. Of particular clinical relevance, as ossification event is defined as the midpoint between two consecutive stages, a proper identification event requires serial radiographs. The main limitation raised by the use of single stages resides in the concept that these stages have variable duration [35, 47, 55, 56] as has been seen for the HWM [5, 55], MPM [37], and CVM [47, 56] methods, making the prediction of the imminent growth spurt less reliable. Therefore, the exact determination of the imminent growth spurt would require closer monitoring of the ossification event, that is, longitudinal recordings, rather than being based on a single stage. This aspect is of further relevance considering that fine transitional changes in the hand and wrist or cervical vertebral morphology may be responsible for determining a pubertal or nonpubertal stage. According to these concepts, longitudinal studies on the capabilities of the different indicators in the identification of the mandibular growth peak (or pubertal growth spurt) are to be preferred over cross-sectional ones. From a clinical standpoint, whenever possible, serial monitoring should be preferred over growth prediction based on single staging.

### 2.2. Correlation Analysis versus Diagnostic Reliability

In spite of the huge number of studies on growth indicators and pubertal growth spurt, the diagnostic reliability of any of the growth indicators in the identification of the peak in standing height or mandibular growth on an individual basis is yet undetermined. Of note, correlations between parameters do not necessarily imply diagnostic accuracy [57, 58].

One of the reasons underlying this noteworthy lack of data may reside in the difficulty of obtaining diagnostic parameters, such as sensitivity, specificity, and accuracy, from longitudinal data in a subset of selected subjects all with a predetermined condition (mandibular growth peak) or a diagnostic outcome (a given HWM/CVM stage). However, the identification of a mandibular growth peak requires longitudinal data, and it is defined as the greatest growth interval [21, 37].

To overcome such limitations, a recent study [21] using already published data on the CVM method [49] has introduced a simple procedure to derive data on diagnostic reliability in the case of longitudinal recordings of growth indicators and mandibular growth. In particular, individual CVM stages and increments in mandibular growth recorded longitudinally were analysed in a group of subjects according to the different predetermined annual (chronological) age intervals. Therefore, a full diagnostic reliability analysis, including sensitivity, specificity, positive and negative predictive values (PPVs and NPVs), and accuracy, of a given CVM stage in the identification of the mandibular growth peak could be carried out within each age interval group. To date only limited longitudinal studies reported on the diagnostic reliability of the CVM [21] and MPM [37] methods in the identification of the mandibular growth peak. Therefore, longitudinal studies reporting diagnostic reliability should be preferred over investigations using bivariate correlations [59, 60] or even multiple regression analyses [61, 62].

### 2.3. Definition of Total Mandibular Length

In several studies on the reliability of growth indicators [34, 35, 63–66] or on the efficiency of functional treatment for Class II malocclusion [17, 26, 67, 68] (see below), the landmark Articulare (Ar) was used instead of the landmark Condylion (Co) to assess the posterior end-point of the mandible. The Ar is defined as the point of intersection of the images of the posterior border of the ramal process of the mandible and the inferior border of the basilar part of the occipital bone [69]. The problem with Ar is that it is not an anatomical landmark that pertains to the mandible exclusively. On the other hand, the landmark Ar has the advantage of being more easily identified as compared to the Co. Even though a previous study [70] reported close correlation between the Ar-Pogonion (Pog) and Co-Pog distances on a sample of 60 cases; other evidence [71, 72] suggested the use of the point Co over Ar as being more reliable in terms of mandibular growth recording. In particular, the posture of the mandible might also affect the position of Ar [71]. Yet repeatability analysis on a cross-sectional sample [70] does not provide evidence that, in a longitudinal analysis, increments in mandibular length (as Ar-Gn and Co-Gn or Ar-Pog and Co-Pog) would yield overlapping patterns of mandibular growth peaks (which are mostly used to validate growth indicators). Therefore, future data are warranted to fully elucidate whether the different landmarks may be used indifferently.

## 3. Hand and Wrist Maturation Method

**Figure 1:**
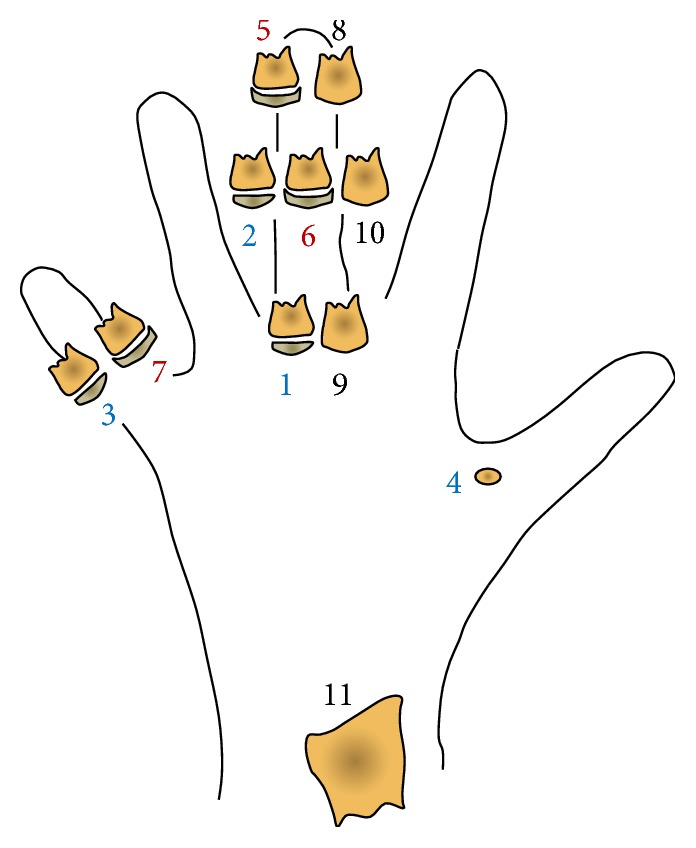
Diagram of the stages of the hand and wrist maturation (HWM) method according to Fishman [35]. The method is also referred to as skeletal maturity assessment (SMA). Blue, prepubertal stages; red, pubertal stages; black, postpubertal stages. See Table 1 for details. Modified from Fishman [35] with permission.

The use of the hand and wrist bones for the assessment of skeletal maturity has initially been reported by Todd [73] followed by others [30, 74, 75]. In particular, all of these methods were based on the assessment of a skeletal age (in years) according to specific ossification events of the hand and wrist. Subsequently, such individual skeletal age had to be compared with reported norms. For reasons listed below, stage-based procedure for the hand and wrist maturation has been added. Among the different stage-based HWM methods [32, 34, 35, 65], the most used nowadays both in research and clinical practice is likely to be that proposed by Fishman [35], also known as skeletal maturation assessment (SMA). Details of the 11-stage HWM method according to Fishman [20] are summarized in Table 1 and shown in Figure 1, while main longitudinal investigations in relation to mandibular growth in untreated subjects without major malocclusion are summarized in Table 2.

### 3.1. Current Evidence

All the published longitudinal studies on the HWM methods and mandibular growth peak included Caucasian [35, 53, 65] and Australian aborigine [34, 76] subjects, and none reported a specific diagnostic reliability analysis. Tofani [65] reported that onset of fusion of distal phalanges are good predictors of mandibular growth peak; however, this study included only females. The study by Grave [34] also reported moderate significant correlations of the hand and wrist maturation with mandibular growth peak for both females and males. A further study by Grave and Brown [76] on the same sample reported previously [34], investigating the HWM method with standing height, reported that peak height velocity would occur up to 3 and 6 months later, in males and females, respectively, of the attainment of the third finger middle phalanx (MP3) stage G (corresponding to the SMI6, See Table 1). In the HWM method according to Fishman [35], peak in mandibular growth (as Ar-Gn) would occur in stage 6 and 7 for females and males, respectively [35]. Further studies correlated this HWM method with standing height [32, 54, 76]. Similarly, the study by Mellion et al. [53] reported for the HWM according to Fishman [35] a moderately strong or weaker relationships in males and females, respectively. In particular, the HWM method assessments had consistently lower errors than either mean chronologic age or CVM method in the identification of both the peaks in standing height and mandibular length [53].

Of note, a previous longitudinal study [77] compared the skeletal age of the whole HWM method (according to Todd [73] and Greulich and Pyle [30]) with specific ossification events of the first, second, and third finger, referred to as the three-finger maturation assessment. As a result, the three-finger maturation assessments were shown to mature in slight advancement than the whole HWM assessments. However, this study [77] was based on correlation analyses and differences in skeletal age between methods, lacking a true diagnostic analysis [78] of concordance or measurement of agreement [79].

### 3.2. Current Controversies

The Greulich and Pyle method [30] and other similar methods [73–75] have been criticized in that it may be difficult to set a reference standard, because of the differential rate of maturation in different bones across individuals of the same population or across different population [54, 80]. For this reason, several standards, that is, norms, have been published for the hand and wrist maturation assessment according to the population of interest. For more detail, see Greulich and Pyle [30] and Todd [73] for white American subjects, Sutow and Ohwada [74] for Japanese subjects, and Tanner and Whitehouse [75] for British subjects. However, such norms are not always available for each population, while another important issue relates to the secular trends, with successive generations becoming taller and reaching puberty at earlier stages [81, 82]. Therefore, the staging of skeletal maturity by describing specific ossification events on the hand-wrist radiograph [32, 34, 35, 53, 65, 66, 83, 84] may be a valid tool as being more independent of differences among populations and secular trends and availability of published standards [80]. The methods based on ossification events [32, 34, 35] might thus be considered to have a wider clinical applicability.

### 3.3. Clinical Implication

Even though the number of studies correlating the HWM methods with mandibular growth peak is limited (Table 2), all of these investigations concluded that these methods may be useful in clinical practice. Therefore, the use of the HWM method may be recommended for planning treatment timing. In spite of this favorable evidence, the HWM method has a main disadvantage residing in the need of an additional film, with consequent increased radiation exposure of the whole hand and wrist. This aspect would prevent a serial recording to monitor closely the ossification events, limiting the diagnosis that has to rely on single stages.

## 4. Third Finger Middle Phalanx Maturation Method

**Figure 2:**
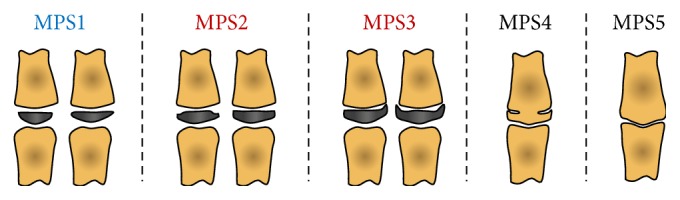
Diagram of the improved third finger middle phalanx maturation (MPM) method according to Perinetti et al. [37]. Blue, prepubertal stages; red, pubertal stages; black, postpubertal stages. See Table 3 for details. Modified from Perinetti et al. [37] with permission.

Previous studies reported above on the HMW methods [34, 54, 76, 85] provided an indication of the possibility for the third finger middle phalanx maturation to the used alone as an indicator of skeletal maturity. Close concurrence of the attainment of MP3 stage G with the peak height velocity has been reported for both males and females [54, 85]. Similar results were seen when correlating the third finger middle phalanx maturation with mandibular growth peak [35, 76]. Therefore, the use of the sole third finger middle phalanx for a maturational method has been proposed [36, 38, 86–88]. This third finger middle phalanx maturation (MPM) method [37, 78] would thus have the advantage of an easy interpretation of the stages, without double contours or superimposition by other structures. Details of a 5-stage MPM method according to Perinetti et al. [37] are summarized in Table 3 and showed in Figure 2, while the only longitudinal investigation [37] in relation to mandibular growth in untreated subjects without major malocclusion is summarized in Table 4.

### 4.1. Current Evidence

All of previous investigations [36–38, 78, 86–88] suggested the use of the MPM method in clinical practice. The main advantage of the MPM method resides in the minimal radiation exposure that would allow close monitoring of the ossification events by longitudinal recordings. Therefore, ideal timing of treatment in individual patients may be identified more precisely as compared to when information comes from single recording, as for the case of the HWM and CVM methods. Finally, the MPM method is of easy execution and interpretation and may be performed in any clinical setting with minimal instrumentation. In spite of the potential clinical advantages offered by the MPM method, current evidence is still little. The present investigations [36, 38, 78, 86–88] are limited by the cross-sectional designs in which the MPM method was analyzed in correlation [36, 38, 86–88] or in diagnostic agreement [78] with the CVM method. Indeed, such analyses do not prove the diagnostic reliability of the method in the identification of the pubertal/mandibular growth peak. The results for the recent longitudinal study [37] on diagnostic reliability (Table 4) showed that the MPM stage 2 (MPS2) precedes the mandibular growth spurt, which is generally concomitant of MPS3. However, even though the overall diagnostic accuracy of 0.91 was satisfactory, the overall positive predictive value was 0.73, thus meaning that false positives may be encountered. This evidence was mainly due to the duration of the MP2 that in some cases lasted for 2 years and it was more evident in the older age groups. Again, the following of the ossification events should be preferred instead of basing growth prediction on single stages [54].

### 4.2. Clinical Implications

Although further investigations are needed, the MPS2 and MPS3 may be considered to be associated with the onset and maximum mandibular growth peak, respectively, in most of the subjects, and may therefore be used for planning treatment timing for functional treatments especially for skeletal Class II malocclusion [4]. According to the minimal radiation exposure, longitudinal monitoring is recommended to follow closely the ossification events. Finally, a combinational use of the MPM method with a further noninvasive indicator of pubertal growth spurt, that is, standing height, especially in the older adolescents, might increase diagnostic reliability [37].

## 5. Cervical Vertebral Maturation Method

**Figure 3:**
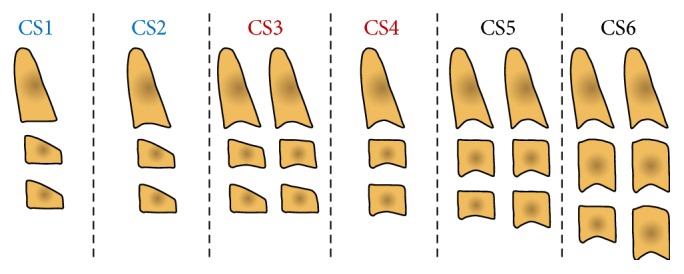
Diagram of the stages of the most common cervical vertebral maturation (CVM) method according to Baccetti et al. [20]. Blue, prepubertal stages; red, pubertal stages; black, postpubertal stages. See Table 5 for details.

The CVM method was initially proposed by Lamparski [39] and then modified by others [20, 33, 46, 49]. In this procedure, the shape of the first cervical vertebrae is analyzed to carry out information on the different growth phase of the subject. In particular, the original method by Lamparski [39] uses vertebrae that can be obscured by the thyroid collar and relied on interstage comparisons, while the subsequent variants of the CVM method [20, 33, 46, 49] were less or not dependent on interstage comparisons. The most common CVM methods are the variants proposed by Hassel and Farman [33] and Baccetti et al. [20], where mandibular growth peak has been reported to occur between stages 3 and 4 [20, 21, 46, 49]. Among the main advantages of the CVM method is the fact that it does not require supplementary radiographic exposure, as for the HWM method, since lateral head film is usually available as a pretreatment record. Details of the 6-stage CVM method according to Baccetti et al. [20] are summarized in Table 5 and shown in Figure 3, while main studies in relation to mandibular growth in untreated subjects without major malocclusion are summarized in Table 6.

### 5.1. Current Evidence

According to previous evidence [20, 46, 49], maturation of the cervical vertebrae occurs in females earlier than in males. Ideally, CVM stages from 2 to 4 should have precise durations in a way that interventions may be easily planned on a basis of a single lateral head film. In this regard, the duration of each CVM stage from 2 to 5 has been reported to last 1 year according to Franchi et al. [49] (Table 6), while little data has been reported to date on the individual durations of the CVM stages [47, 53, 61]. This does not allow the easy planning of the timing of intervention based only on a single lateral head film. A further study by Ball et al. [47] (Table 6) reported very different results with the CVM stages 2, 3, 4, and 5 with longer durations of about 1.9, 1.8, 3.8, and 2.9 years, respectively. On the contrary, another longitudinal investigation [61] reported mean duration of about 1 year for the CVM stages from 2 to 4. Interestingly, longer and shorter CVM stages 3 to 4 intervals have been reported for Class III [89] and Class II subjects [90], respectively, as compared to that of Class I subjects. However, these studies [89, 90] were limited by their cross-sectional design not allowing the detection of any individual variation in the duration of single CVM stages. Longitudinal studies correlating facial growth patterns with duration of CVM stages are still missing.

Many previous studies were limited to the correlation analyses between the different CVM and HWM methods [33, 36, 48, 59, 86–88, 91, 92] with no information on the mandibular growth (or standing height) peak; other studies were limited to the longitudinal investigation of the cervical vertebral maturational changes [56] or investigated the potential of the CVM method to detect postpubertal mandibular growth [50]. A further investigation [62] was focused on the capability of the CVM method to predict the total amount of mandibular growth from prepubertal to postpubertal phases, irrespective of timing of pubertal growth peak [93], and it included exclusively Class II female subjects, where mandibular growth peak has been shown to be minimal or absent [23].

However, as for the HWM method, the most relevant information may be derived from longitudinal studies investigating the capabilities of these methods in detecting the mandibular growth peak, possibly in individual subjects. Previous studies on the CVM method and mandibular growth peak have reported contrasting results of negligible [47, 48, 53, 61, 62] and noteworthy [49, 52, 64, 66] correlations. Interestingly, only few studies [21, 47, 49, 52, 53, 61, 64, 94] (Table 6) correlated the CVM method (as stage system) with mandibular growth under longitudinal monitoring. According to this evidence, a total of five studies [21, 49, 52, 64, 94] reported mandibular growth peak to occur during stages 3 and 4, and four [21, 49, 64, 94] of them recommended the use of the CVM method in treatment planning. One study [21], however, used the same sample of Franchi et al. [49] from which the CVM method was derived. The remaining three studies [47, 53, 61] failed to detect a significant correlation between the CVM and mandibular growth peak and did not recommend the method for treatment planning.

### 5.2. Current Controversies

When reporting on the CVM method, the different variants of the method [20, 33, 46, 48, 84] have to be taken into account and results should be limited to the investigated methods or parameters [95]. Significant differences in study designs, cephalometric recordings, and data analysis have to be taken into account when dealing with clinical usefulness of the CVM method. For instance, apart from the study [21] using the same sample reported by Franchi et al. [49] (Table 6), the only investigation [66] that has reported on the diagnostic capability of the CVM method in the identification of the mandibular growth peak used receiver operating characteristics curves. However, this study [66] was based on a cross-sectional sample and it was limited to the analysis of the area under the curve, which is not enough to describe in full the diagnostic reliability of the method. Therefore, conclusions on the diagnostic reliability of the CVM method in the identification of the mandibular growth peak have conjecturally been based on difference among groups/stages [47, 49, 52, 64, 94], regression analyses [61], or other analyses missing diagnostic capabilities [53].

Another relevant issue when dealing with the CVM method resides in its repeatability. The method has been reported to have poor repeatability [96, 97]. Although this limitation may be avoided by proper training [98], poor repeatability has been seen even in studies correlating the CVM method with mandibular growth [62], while longitudinal investigations herein considered (Table 6) reported no information [52, 53, 64, 94] or good to high repeatability [47, 49, 61] in the CVM stage assignment. Finally, when assigning the CVM stage, it has been suggested that exceptional cases, that is, cases outside the reported norms, may exist [98] and this may be responsible for doubtful interpretation and poor reproducibility.

### 5.3. Clinical Implications

As for the HWM method, the CVM methods require films that are usually available as a pretreatment record, while optimal treatment timing is to be delayed for an undermined term after the diagnosis. Therefore, further reevaluation of the growth phase needs a reexecution of a lateral head film, which would not be indicated. Moreover, the cervical vertebrae might be partially covered by the protection collar, which would be necessary to reduce radiation exposure [99]. Apart from this consideration, the use of the CVM method requires proper training in stage assignment and knowledge of exception cases [98]. Moreover, variability in duration of the CVM stages 2 to 4 [47, 56] has been taken into account and functional treatment requiring the inclusion of the mandibular growth spurt in the active treatment period should last until attainment of CS5 [21]. Future longitudinal studies on diagnostic reliability of the CVM method in the identification of the mandibular growth peak are still necessary to fully elucidate the clinical usefulness of the method.

## 6. Dental Maturation Method

**Figure 4:**
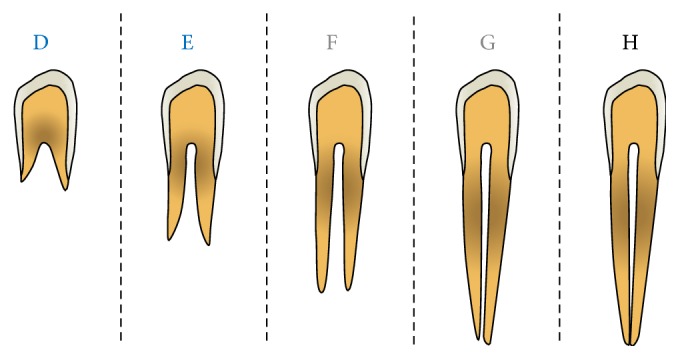
Diagram of the stages of the most common dental maturation method according to Demirjian et al. [40]. Only the stages D to H are represented due to their relevance with the circumpubertal growth phase. In molars, the distal root should be considered in assessing the G and H stages. Blue, prepubertal stages; grey, any stage; black, postpubertal stages. See Table 7 for details.

Dental maturity can be assessed by the exfoliation of deciduous teeth, such as the second molars [100], phases of dentition [101], dental emergence [5, 32], or calcification stages through the evaluation of tooth formation [40]. Calcification stages of the teeth can be carried out on panoramic radiographs that are routinely used for different purposes, with mandibular teeth preferred over maxillary ones being less subjected to superimpositions from other skeletal structures. Even intraoral radiograph may be used with minimal irradiation to the patient. Therefore, dental maturation has been proposed as a further useful method for assessing the growth phase in individual subjects [31]. The most common method used for scoring dental maturation is the one described by Demirjian et al. [40]. This method has the advantage of using relative values of the root formation to the crown height, rather than absolute lengths. Foreshortened or elongated projections of developing teeth will not affect the reliability of this assessment [40]. Details of the dental maturation method according to Demirjian et al. [40] are summarized in Table 7 and shown in Figure 4, while main cross-sectional studies of diagnostic reliability using the HWM or CVM methods in untreated subjects without major malocclusion are summarized in Table 8.

### 6.1. Current Evidence

The period corresponding to the exfoliation of the deciduous second molars has been advocated as favorable for the beginning of a one-phase orthodontic treatment in growing subjects [102]. However, as previously reported [100], the exfoliation of the deciduous second molars has no significant relationship with the onset of the pubertal growth spurt (Table 8). Similarly, the assessment of the phase of dentition (as deciduous, early mixed, mixed, and permanent) is a simple procedure and has been used to assess the effects of different treatment timing in Class II patients [103]. However, the only study [101] on diagnostic reliability (Table 6) reported that neither the early mixed nor the mixed dentition phases are valid indicators of the pubertal growth spurt. Therefore, the use of the exfoliation of the deciduous second molar or phases of dentition is not recommended for treatment planning. Similarly, dental emergence has also been reported to be poorly correlated with pubertal growth spurt [5, 32]. Regarding dental calcification stages, high correlations with skeletal maturity have been reported by most of the investigations performed to date using the CVM [31, 60, 86, 104–109], HWM [106, 110, 111] or MPM [86, 112] methods. As a consequence, most of the studies have proposed the staging of dental maturation as a reliable indicator of the individual skeletal maturity, which has major diagnostic implications [31, 60, 106–109, 111, 113–117]. On the contrary, other studies [104, 105, 110, 112] (Table 8) including a meta-analysis [118] reported a very limited clinical usefulness of dental maturation in the identification of the pubertal growth spurt.

### 6.2. Current Controversies

The apparent inconsistency among all the current investigations on dental and skeletal maturation (all cross-sectional) resides in the use of proper diagnostic reliability analysis. The present evidence on diagnostic reliability [104, 105, 110, 112] has revealed that the conclusions reported in previous investigations based on correlational analyses [31, 60, 106–109, 111, 113–117] were not actually supported by the results obtained in those studies. The few exceptions seen for early dental developmental stages, which were reliable in the identification of the prepubertal growth phase [105, 110, 112, 118], would have poor clinical meaning since early mixed and intermediate mixed dentition may be used instead for the same purpose [5, 32, 101]. Longitudinal studies on the diagnostic reliability of dental maturation, mainly as calcification stages, in the identification of the mandibular growth peak are still missing.

### 6.3. Clinical Implications

Irrespective of the mandibular tooth, none of the dental maturation stages may be reliably used to identify in individual subjects the pubertal growth spurt (Table 8). Other indicators remain preferable for the determination of the growth phase in individual growing patients [118].

## 7. Other Indicators

### 7.1. Standing Height

Standing height has been used as an indicator of the pubertal growth spurt from several decades ago [3, 5, 119, 120]. This procedure requires several measurements of standing height repeated at regular intervals to construct an individual curve of growth velocity and has the advantage of being noninvasive. The peak in standing height has been reported to precede [3, 119] or to be in concurrence [120, 121] with the peaks in facial bones growth. Other evidence reported that standing height had little predictive value in determining the growth profile of any of the mandibular parameters except for Ar-Pog for females [63]. Mandibular growth peak has been seen to occur in concurrence with or slightly after the peak in standing height for males and females, respectively [35]. In a more recent investigation [53], the peak in stature had a shorter duration and tended to occur a few months before that of the face and mandible. Although all of these investigations [3, 5, 32, 34, 49, 53, 119–121] reported a satisfactory degree of correlation between the standing height and mandibular growth, data on diagnostic reliability of standing height peak in the identification of the mandibular growth peak has been reported only in one study [21]. In particular, a variable diagnostic accuracy (between 0.61 and 0.95) was seen for the standing height peak in the identification of the mandibular growth peak (as greatest annual increments in Co-Gn or in mean value between Co-Gn and Co-Go) [21]. From a clinical perspective, therefore, the recording of standing height may be useful, especially in conjunction with other radiographical indicators.

### 7.2. Chronological Age

Several investigations [32, 35, 53, 122, 123] reported that the average ages at the onset and peak of pubertal growth in stature are about 12 and 14 years in boys and 10 and 12 years in girls. However, a noteworthy variability was also seen when pubertal growth spurt was defined as standing height peak [21, 35, 54, 63, 65, 76, 84, 92] or mandibular growth peak [37, 49, 52, 64]. To date, only one cross-sectional study [124] reported on diagnostic performance of chronologic age in the identification of the pubertal growth phase (according to the CVM method [20]). In males, age up to 9 years can reliably identify a prepubertal stage of skeletal development, and in females an age of at least 14 years can reliably identify a postpubertal stage. In both males and females, chronologic age could not reliably identify the onset of the pubertal growth phase [20]. Therefore, in spite of the simplicity of the method, its clinical applicability as an indicator of the onset of the pubertal growth spurt in the individual patient is limited [20, 21, 32, 37]. On the contrary, the study by Mellion et al. [53] reported that chronological age would have only a slightly greater error, as compared to that of the HWM according to Fishman [35], in the identification of the mandibular growth peak and it is therefore recommended for the treatment planning. However, this only evidence [53] derived from an old sample (Tables 2 and 6) has to be confirmed by further investigation, especially considering that onset of puberty can be influenced by several factors including genetics, ethnicity, nutrition, and socioeconomic status [82] responsible for a secular trend [81].

### 7.3. Menarche and Voice Change

Menarche usually occurs immediately after [123, 125] or 1 year after the pubertal growth spurt [5, 126]. According to other evidence [65], menarche would occur after the mandibular growth peak in the early- and average-maturing girls, while in late-maturing girls it may generally occur before the mandibular growth peak. However, late-maturing girls would represent a minority of the population rendering this indicator useless [65]. Similarly, in boys, the voice change occurs during or after the pubertal growth spurt [54, 125]. Therefore, these two indicators are not usable in planning treatment timing in orthodontics.

### 7.4. Biomarkers

The use of biomarkers has been proposed very recently as a new aid in assessing individual skeletal maturity, with the advantage of being related to the physiology of the patient and of avoiding the use of radiations. The very scarce data reported to date include molecular constituents from the serum, such as insulin-like growth factor I (IGF-I) [42, 43, 127], or from the gingival crevicular fluid (GCF), such as alkaline phosphatase (ALP) [41, 44] or total protein content [45]. These studies reported increased levels of the investigated biomarkers during the pubertal growth spurt as compared to the prepubertal and postpubertal growth phases [41–44, 127] with the exception of the GCF total protein content [45]. However, these studies followed cross-sectional designs and used the CVM method to assess pubertal growth phase [41–45], with one exception where a sample of 25 subjects was followed longitudinally in their mandibular growth [127]. Of particular interest are the biomarkers from the GCF, since its sampling involves a very simple, rapid, and noninvasive procedure that can be performed in a clinical setting. However, even though dental permutation has been reported not to influence significantly the GCF ALP activity [128], variability among the subjects and method errors [129] have to be taken into account. Moreover, optimal gingival conditions without plaque accumulation or clinically evident inflammation is necessary as the GCF ALP activity reflects local tissue inflammation [130]. Future studies on the diagnostic reliability of these biomarkers in the identification of the pubertal growth spurt or mandibular growth peak are warranted.

## 8. Efficiency of Functional Treatment for Skeletal Class II Malocclusion

### 8.1. Current Evidence

Herein, to report and evaluate critically current evidence on functional treatment for skeletal Class II malocclusion, data from most recent meta-analyses has been reviewed. Several meta-analyses on the efficiency of functional treatment for Class II malocclusion (skeletal or not) [11–19, 131–133] have been published reporting contrasting results. Some evidence has shown how functional treatment for skeletal Class II malocclusion may be effective in terms of mandibular elongation [17, 18, 132, 133] or dentoalveolar compensation [15, 16]. On the contrary, other evidence reported minimal effects for such treatment [11, 13, 131]. The reason for this apparent inconsistency might reside in the different interventions performed [19, 134], in the large variation in individual responsiveness to functional treatment [17, 18] in conjunction with the absence of an analysis of potential prognostic factors [135], type of appliance [14, 17, 18, 131, 132], and patient's compliance for the removable appliances. Most recent meta-analyses [14–18, 131, 133] including untreated matched Class II control subjects with contrasting outcomes have been herein summarized (Table 9). In particular, these meta-analyses have been analysed according to the main sources of controversies such as design of clinical trials, definition of Class II malocclusion, and skeletal maturity.

### 8.2. Design of Clinical Trials

When performing clinical trials on the efficiency of functional treatment for Class II malocclusion, a relevant ethical issue relates to the leaving of subjects with relevant malocclusions without orthodontic treatment during the pubertal growth spurt. This issue has limited the execution of randomized clinical trials (RCTs) at this stage of development. Therefore, reviews including exclusively RCTs [11–13] might have been focused mostly on prepubertal subjects, leaving the potential effects of treatment on pubertal patients excluded from the analysis. To date the only exception is for an RCT [24] executed on a group of pubertal patients reporting clinically relevant effects for functional treatment in reducing the entity of the skeletal Class II malocclusion. On the contrary, other most relevant RCTs performed to date included exclusively [27, 29] or mostly [136] prepubertal patients. For this reason, the consideration of controlled clinical trials (CCTs) with reasonable methodological quality has been advocated [137], especially considering that whenever RCTs are not available for meta-analysis, CCTs or observational studies may be used with essentially similar outcomes [138]. In spite of a previous meta-analysis including exclusively RCTs [13], the most recent ones herein summarized included both RCTs and CCTs, although an attempt has been made in several cases to the inclusion of prospective trials over retrospective investigations (Table 9).

### 8.3. Definition of Class II Malocclusion

A clear distinction should be made between skeletal and dentoalveolar Class II malocclusion. Interestingly, clinical trials [27, 29] on the efficiency of functional treatment for Class II malocclusion used overjet (equal or above 7 mm) as the only diagnostic criterion for Class II malocclusion. However, such an overjet as a sole diagnostic parameter has been shown to be not fully reliable in the identification of a skeletal Class II malocclusion [139]. On the contrary, other trials [140, 141] used specific cephalometric parameters to assure the inclusion of skeletal Class II patients. In the meta-analyses herein reported, trials were included according to dental parameters alone [15, 16, 131], to a combination of ANB angle equal to or above 4° in combination with at least half-cusp Class II molar relationship [17, 18], or to nonspecified criteria [14, 133]. Therefore, conclusions on the supplementary mandibular elongation consequent to functional treatment should be limited to those trials including true skeletal Class II patients due to retrognathic mandible [17, 18].

### 8.4. Skeletal Maturity

In spite of the previous evidence suggesting skeletal maturity as a potential prognostic factor in terms of skeletal effects produced by functional treatment in skeletal Class II patients [4, 25, 134, 142], to date few clinical trials have focused on the timing of intervention. The assessment of skeletal maturity, with clear distinction among prepubertal, pubertal, and postpubertal groups, was an inclusion criterion only for 2 meta-analyses [17, 18], while it was not considered for all the others [14–16, 131, 133]. However, information on skeletal maturity, when available, was extracted in most of the meta-analyses (Table 9). Subgroup analysis for the different growth phases (mainly prepubertal versus pubertal patients) was performed in 4 meta-analyses [15–18], even though it was inconclusive in 1 case [15] because of limited data available, while, in another case, prepubertal and pubertal patients were pooled [16]. Of note, meta-analyses in which skeletal maturation was not considered or not analyzable [14, 15, 131] reported minimal effects of dentoalveolar nature, while meta-analyses evaluating specifically [17, 18] or mostly [133] pubertal patients reported clinically relevant effects in terms of mandibular elongation and reduction of the skeletal Class II malocclusion (Table 9).

### 8.5. Other Limitations of the Current Studies

The current investigation on the effects of functional treatment of Class II malocclusion is inherently hampered by other factors [14–18, 131, 133]. For instance, in spite of the use of annualized changes, observational terms may include not only the effective functional treatment, but also variable periods of time of retention or of further management of the dentition. Therefore, skeletal changes might occur not uniformly during the entire observational term skewing the analysis of treatment outcomes [12]. It is hard to avoid heterogeneity of the selected studies because of small sample sizes, inclusion of retrospective trials with historical control groups, and similar skeletal outcomes defined by different cephalometric parameters. Finally, an analysis of the potential responsiveness to treatment according to specific prognostic factors is still not feasible, and current evidence is mostly focused on the short-term effects.

## 9. Concluding Remarks

Current evidence on both the reliability of growth indicators and efficiency of functional treatment for skeletal Class II malocclusion is still controversial and highly heterogeneous. Although no skeletal maturity indicator may be considered to have a full diagnostic reliability in the identification of the pubertal growth spurt or mandibular growth peak, treatment timing according to available indicators (mainly HWM and CVM methods) has yielded more favorable outcomes in terms of mandibular elongation and reduction of the Class II malocclusion. The use of the HWM or CVM methods (or others) may still be recommended for treatment planning, even though large individual responsiveness and dentoalveolar compensations have been reported even in pubertal patients. Future investigation will have to further elucidate the controversies reported herein and follow more robust designs.

## Figures and Tables

**Table 1:** Description of the stages of the hand and wrist maturation (HWM) method according to Fishman [35].

Stage description	Attainment
SMI 1: third finger proximal phalanx, epiphysis as wide as metaphysis	Before the standing height and mandibular growth peaks (prepubertal)
SMI 2: third finger middle phalanx, epiphysis as wide as metaphysis	
SMI 3: fifth finger middle phalanx, epiphysis as wide as metaphysis	
SMI 4: thumb, appearance of adductor sesamoid	

SMI 5: third finger distal phalanx, epiphysis showing capping towards the metaphysis	Generally, at coincidence of the standing height and mandibular growth peaks (pubertal)
SMI 6: third finger middle phalanx, epiphysis showing capping towards the metaphysis	
SMI 7: fifth finger middle phalanx, epiphysis showing capping towards the metaphysis	

SMI 8: third finger distal phalanx, fusion of epiphysis and diaphysis	After the standing height and mandibular growth peaks (postpubertal)
SMI 9: third finger proximal phalanx, fusion of epiphysis and diaphysis	
SMI 10: third finger middle phalanx, fusion of epiphysis and diaphysis	
SMI 11: radius, fusion of epiphysis and diaphysis	

The method is also referred to as skeletal maturity assessment (SMA). SMI, skeletal maturity indicator.

**Table 2:** Main longitudinal studies on the hand and wrist maturation (HWM) method and mandibular growth peak in untreated subjects without major malocclusion.

Study	Sample origin and other information	Sample size and sex distribution/age range	Hand and wrist maturation assessment	Main mandibular parameter(s)	Statistical analysis	Main results	Clinical implications according to the authors
Tofani 1972 [65]	Broadbent-Bolton growth study	20 F/9–18 yrs	Onset of fusion of the first and third finger distal phalanges	Ar-Pog, Ar-Go, Go-Pog	Differences between pre- and postpubertal and correlation analyses	Age of onset of fusion of distal phalanges and that for mandibular growth peak were significantly correlated	Onset of fusion of distal phalanges are good predictors of mandibular growth peak

Grave 1973 [34]	Australian aborigines	36 F, 52 M/8–18 yrs	Custom method	Ar-Pog	Correlation analyses	Some moderate significant correlations were seen for females and males	The HWM method may be useful in clinical practice

Fishman 1982 [35]	Denver Child Research Study and own practice	206 F, 196 M/0–25 yrs	Eleven-stage method (SMIs) according to Fishman [35] (Figure 1)	Ar-Gn	Differences among stages	Maximum growth increments were seen during stages 5–7	The SMIs provide a key to identification of maturation level with important clinical applications

Mellion et al. 2013 [53]	Broadbent-Bolton growth study (a)	50 F, 50 M/8 and 10 yrs at least for females and males, respectively, with 6 to 11 annual recordings	Eleven-stage method (SMIs) according to Fishman [35] (Figure 1)	Co-Gn	Actual age at onset and peak in mandibular growth used as the gold standards against which key ages inferred from SMIs method was compared	The SMIs showed in males and females a moderately strong or weaker relationships, respectively, to the timing for the onset and peak in mandibular growth	The SMIs appear to offer the best indication that peak growth velocity has been reached

Studies using maturation method based on ossification events (stages) are represented. Ar, Articulare; Pog, Pogonion; Go, Gonion; Gn, Gnathion; Co, Condylion; SMIs, skeletal maturation indicators (according to Fishman [35]). *Note.* a: it may include some Class II subjects.

**Table 3:** Description of the stages of the third finger middle phalanx maturation (MPM) method according to Perinetti et al. [37].

Stage description	Attainment
MPS1: epiphysis is narrower than the metaphysis, or epiphysis is as wide as metaphysis but with both tapered and rounded lateral borders. Epiphysis and metaphysis are not fused. Reported as MP3-F [32]	More than 1 year before the onset of the pubertal growth spurt [32] or mandibular growth peak [37]

MPS2: epiphysis is at least as wide as the metaphysis with sides increasing thickness and showing a clear line of demarcation at right angle, either with or without lateral steps on the upper contour. In case of asymmetry between the two sides, the more mature side is used to assign the stage. Reported as SMI2 [35] or as MP3-FG [32]	1 year before the pubertal growth spurt [32] or mandibular growth peak [37]

MPS3: epiphysis is either as wide as or wider than the metaphysis with lateral sides showing an initial capping towards the metaphysis. In case of asymmetry between the two sides, the more mature side is used to assign the stage. Epiphysis and metaphysis are not fused. Reported as SMI6 [35] or as MP3-G [32]	At coincidence of the pubertal growth spurt [32] or mandibular growth peak [37]

MPS4: epiphysis begins to fuse with the metaphysis although contour of the former is still clearly recognizable. The capping may still be detectable. Reported as MP3-H [32]	After the pubertal growth spurt [32] or mandibular growth peak [37]

MPS5: epiphysis is totally fused with the metaphysis. Reported as SMI10 [35] or as MP3-I [32]	At the end of the pubertal growth spurt [32]

**Table 4:** Main longitudinal studies on the third finger middle phalanx maturation (MPM) method and mandibular growth peak in untreated subjects without major malocclusion.

Study	Sample origin and other information	Sample size and sex distribution/age range	Middle phalanx maturation assessment	Main mandibular parameter	Statistical analysis	Main results	Clinical implications according to the authors
Perinetti et al. 2016 [37]	Burlington growth study	15 F, 20 M/9–16 yrs	Five-stage custom method (Figure 2)	Co-Gn	Diagnostic performance	Stage 2 had a satisfactory but variable accuracy in the identification of imminent mandibular growth peak	The MPM method may be useful in treatment timing

Co, Condylion; Gn, Gnathion.

**Table 5:** Description of the stages of the most common cervical vertebral maturation (CVM) method according to Baccetti et al. [20] with corresponding codes.

Stage description	Attainment
CS1: lower borders of the second, third, and fourth vertebrae (C2, C3, and C4) flat and the bodies of C3 and C4 trapezoid in shape	At least 2 years before the pubertal growth spurt

CS2: only the lower border of C2 with concavity and the bodies of C3 and C4 trapezoid	About 1 year before the pubertal growth spurt

CS3: lower borders of C2 to C3 with concavities and the bodies of C3 and C4 either trapezoid or rectangular horizontal in shape	At coincidence of the ascending portion of the pubertal growth spurt

CS4: lower borders of C2 to C4 with concavities and the bodies of both C3 and C4 both (or at least one, [a]) rectangular horizontal	At coincidence of the descending portion of the pubertal growth spurt

CS5: lower borders of C2 to C4 with concavities and at least one or both of the bodies of C3 and C4 squared.	About 1 year after the pubertal growth spurt

CS6: lower borders of C2 to C4 with concavities and at least one or both of C3 and C4 rectangular vertical	At least 2 years after the pubertal growth spurt

**Table 6:** Main longitudinal studies on the cervical vertebral maturation (CVM) method and mandibular growth peak in untreated subjects without major malocclusion.

Study	Sample origin and other information	Sample size and sex distribution/age range	Cervical vertebral maturation assessment	Main mandibular parameter(s)	Statistical analysis	Main results	Clinical implications according to the Authors
O'Relly and Yanniello 1988 [64]	Broadbent-Bolton growth study	13 F/9–15 yrs	Six-stage Lamparski's standards	Ar-Pog, Ar-Go, Go-Pog	Differences among stages	Stages 1–3 occurring the year preceding the peak in most cases	The CVM can be used to assess timing of mandibular growth

Franchi et al. 2000 [49]	Michigan growth study	15 F, 9 M/7–16 yrs	Six-stage modified Lamparski's standards	Co-Gn, Co-Goi, Goi-Gn	Differences among stages	Total mandibular length showed the greatest significant increment between stages 3 and 4	The CVM is a valid method for the evaluation of skeletal maturity and mandibular growth peak

Gu and McNamara 2006 [52]	Part of the Mathews and Ware implant sample [143]	13 F, 7 M/≈7–17 yrs	Six-stage method according to Baccetti et al. [20] (Figure 3)	Co-Gn, Co-Go, Go-Me	Differences among stages	Peak in mandibular length observed between stages 3 and 4	Not reported

Chen et al. 2010 [108]	Research Centre of Craniofacial Growth and Development at Beijing University.	55 F, 32 M/8–18 yrs	Four-stage quantitative method [94]	Ar-Gn, Ar-Go, Go-Gn	Differences among stages (as absolute and relative growth increment)	Maximum growth increments were seen during stage II (b) with relative increments more consistent than absolute ones	Use of the quantitative CVM method is recommended for treatment planning

Ball et al. 2011 [47]	Burlington growth study	90 M/9–18 yrs	Six-stage method according to Baccetti et al. [20] (Figure 3)	Ar-Gn	Differences among stages in groups of advanced, average, and delayed maturation	Mandibular growth peak occurred mainly during stage 4 (which lasted 3.8 yrs)	The CVM method cannot predict the onset of the mandibular growth peak

Mellion et al. 2013 [53]	Broadbent-Bolton growth study (a)	50 F, 50 M/8 and 10 yrs at least for females and males, respectively, with 6 to 11 annual recordings	Six-stage method according to Baccetti et al. [20] (Figure 3)	Co-Gn	Actual age at onset and peak in mandibular growth used as the gold standards against which key ages inferred from CVM method was compared	The CVM stages showed only a weak to moderate relationship to the timing for the onset and peak in mandibular growth	Use of the CVM method is not recommended for treatment planning

Gray et al. 2016 [61]	Burlington growth study	12 F, 13 M/10–16 yrs	Six stage method according to Baccetti et al. [20] (Figure 3)	Ar-Gn	Mixed linear regression	Mandibular length changes were not significantly associated with CVM stages	The CVM method does not accurately identify the mandibular growth peak

Perinetti et al. 2016 [21]	Same as Franchi et al. [49]	Same as Franchi et al. [49]	Same as Franchi et al. [49]	Co-Gn, Co-Goi, mMG	Diagnostic performance	Stages 3-4 have variable diagnostic accuracy in the identification of mandibular growth peak	The CVM can be used in clinical practice. Limitations due to the use of the same sample from which the method was derived

Studies using maturation method based on ossification events (stages) are represented. Ar, Articulare; Pog, Pogonion; Go, Gonion; Co, Condylion; Gn, Gnathion; Goi, Gonion intersection; mMG, mean mandibular growth ([Co-Gn + Goi-Gn]/2). *Note*. a: it may include some Class II subjects; b: stage II equivalent to stage 3 in the 6-stage CVM method.

**Table 7:** Description of the stages of the most common dental maturation method according to Demirjian et al. [40].

Stage description	Attainment
*Stage D*. When (1) the crown formation is complete down to the cementoenamel junction; (2) the superior border of the pulp chamber in the single-root teeth has a definite curved form, with it being concave towards the cervical region; the projection of the pulp horns, if present, gives an outline shaped like the top of an umbrella and (3) the beginning of root formation is seen in the form of a spicule	Canine, premolars, and second molar before the pubertal growth spurt [28, 57, 110, 112]

*Stage E*. When (1) the walls of the pulp chamber form straight lines, the continuity of which is broken by the presence of the pulp horn, which is larger than in the previous stage and (2) the root length is less than the crown height	Mostly, canine and first premolar before the pubertal growth spurt [28, 57, 110, 112]

*Stage F*. When (1) the walls of the pulp chamber form a more or less isosceles triangle, with the apex ending in a funnel shape and (2) the root length is equal to or greater than the crown height	Sometimes, canine before the pubertal growth spurt [28, 57, 112]

*Stage G*. When the walls of the root canal are parallel and its apical end is still partially open	Canine, premolars, and second molar before, during, and after the pubertal growth spurt [28, 57, 110, 112]

*Stage H*. When (1) the apical end of the root canal is completely closed and (2) the periodontal membrane has a uniform width around the root and the apex	Second molar after the pubertal growth spurt [28, 57, 112]

Only stages D to H are summarised due to their relevance with the circumpubertal growth phase. In molars, the distal root is considered in assessing the stage [40]. Only results from studies reporting diagnostic reliability analysis are shown regarding the moment of attainment of the different stages for mandibular teeth.

**Table 8:** Main cross-sectional studies on the dental emergence and dental maturation method according to Demirjian et al. [40] and hand and wrist or cervical vertebral maturation in untreated subjects without major malocclusion.

Study	Sample size and sex distribution/age range	Dental maturation assessment	Skeletal maturation assessment	Statistical analysis	Main results	Clinical implications according to the authors
Tassi et al. 2007 [100]	428 (a)	Exfoliation of the deciduous second molars	CVM, 6-stage method according to Baccetti et al. [20]	Sensitivity, specificity, PPV, positive LHR	No significant relationship between the moment of exfoliation of deciduous second molars and the onset of the pubertal growth spurt	Not recommended for treatment planning

Franchi et al. 2008 [101]	500 F, 500 M/≈6–14 yrs	Early mixed, mixed, late mixed, and permanent	CVM, 6-stage method according to Baccetti et al. [20]	Sensitivity, specificity, PPV, positive LHR	Mixed dentition and early permanent dentition are not valid indicators for the onset of the pubertal growth spurt	Not recommended for treatment planning

Perinetti et al. 2012 [57]	208 F, 146 M/6.8–17.1 yrs	Mandibular teeth	CVM, 6-stage method according to Baccetti et al. [20]	Sensitivity, specificity, PPV, NPV, accuracy, positive LHR	Dental maturation assessment is reliable in the identification of prepubertal and postpubertal growth phases	Not recommended for treatment planning

Perillo et al. 2013 [28]	192 F, 108 M/6.8–17.1 yrs	Mandibular canine and second molar	CVM, 6-stage method according to Baccetti et al. [20]	Sensitivity, specificity, PPV, NPV, accuracy, positive LHR	Combined canine and second molar maturation has little role in the identification of the pubertal growth spurt	Not recommended for treatment planning

Surendran and Thomas 2014 [112]	71 F, 79 M/8–16 yrs	Mandibular teeth	MP3, 6-stage method according to Rajagopal and Kansal [36]	Positive LHR	Dental maturation assessment is reliable in the identification of prepubertal and postpubertal growth phases	Recommended only for planning treatments that need to be performed in prepubertal patients

Cericato et al. 2016 [110]	314 F, 262 M/7–18 yrs	Mandibular teeth	CVM, 6-stage method according to Hassel and Farman [33] and Baccetti et al. [20]	Positive LHR	Dental maturation assessment is reliable in the identification of prepubertal growth phases	Not reported

Only studies reporting diagnostic performance are represented. No longitudinal study has been reported to date. CVM, cervical vertebral maturation; PPV, positive predictive value; LHR, likelihood ratio; NPV, negative predictive value; a, other information not provided.

**Table 9:** Most recent meta-analyses including controlled trials on mandibular effects produced by functional treatment in Class II patients.

Study	Included trials		Appliance	Skeletal maturity			Further notes or results on skeletal maturity	Clinical Implications on functional treatment for Class II malocclusion according to the authors
	Design	Definition of Class II malocclusion		Inclusion criterion	Data extraction	Subgroup analysis		
*Removable appliances*								
Ehsani et al. 2015 [14]	RCTs, CCTs (prospective or retrospective)	Not specified	Twin-block	No	No	No	Not reported	Individual changes were of limited clinical significance, but when combined reached clinical relevance

Koretsi et al. 2015 [15]	RCTs, CCTs (prospective)	A combination of dental and skeletal parameters or only dental parameters	Various	No	Yes	Prepubertal versus pubertal	Comparisons between pubertal and prepubertal inconclusive because of limited data available	Effective, although main effects seem to be mainly dentoalveolar rather than skeletal

Perinetti et al. 2015 [18]	RCTs, CCTs (prospective or retrospective)	ANB > 4° and Class II molar relationship, at least	Various	Yes	Yes	Prepubertal versus pubertal	Annualized supplementary total mandibular elongation was 0.9 mm and 2.9 mm in prepubertal and pubertal patients, respectively.	Effective, with clinically relevant skeletal effects only if performed during the pubertal growth phase

*Fixed appliances*								
Al-Jewair 2015 [131]	RCTs, CCTs (prospective or retrospective)	Molars in at least an end-to-end relationship	MARA	No	Yes	No	Five out of 7 studies included subjects at onset or pubertal growth phase	Effects may be not clinically relevant (although statistically significant)

Perinetti et al. 2015 [17]	RCTs, CCTs (prospective or retrospective)	ANB > 4° and Class II molar relationship, at least	Various, with or without FFAs	Yes	Yes	Pubertal versus postpubertal	Supplementary total mandibular elongation was 2.2 mm and 0.4 mm in pubertal and postpubertal patients, respectively. Little data available on the postpubertal subjects	Effective, with clinically relevant skeletal effects only if performed during the pubertal growth phase

Yang et al. 2016 [133]	CCTs (prospective)	Skeletal Class II	Herbst	No	Yes	No	Most of the subjects were treated during the pubertal growth spurt	Effective, with relevant changes on dental discrepancy and skeletal changes

Zymperdikas et al. 2016 [16]	RCTs, CCTs (prospective)	A combination of dental and skeletal parameters, or only dental parameters	Various	No	Yes	Prepubertal and pubertal (merged) versus postpubertal	Trend towards more favourable changes in the prepubertal and pubertal than in the postpubertal patients although not statistically significant	Effective, although main effects seem to be mainly dentoalveolar rather than skeletal

Meta-analyses published over the last 2 years are reported. Notes: RCTs, randomized clinical trials; CCTs, controlled clinical trials; MARA, mandibular anterior repositioning appliance; FFAs, full fixed appliances. Results are limited to the short-term effects.

## References

[1] Björk A. (1963). Variations in the growth pattern of the human mandible: longitudinal radiographic study by the implant method. *Journal of Dental Research*.

[2] Harris J. E. (1962). A cephalometric analysis of mandibular growth rate. *American Journal of Orthodontics*.

[3] Nanda R. S. (1955). The rates of growth of several facial components measured from serial cephalometric roentgenograms. *American Journal of Orthodontics*.

[4] Petrovic A., Stutzmann J., Lavergne J., Carlson D. S. (1990). Mechanism of craniofacial growth and modus operandi of functional appliances: a cell-level and cybernetic approach to orthodontic decision making. *Craniofacial Growth Theory and Orthodontic Treatment*.

[5] Björk A., Helm S. (1967). Prediction of the age of maximum puberal growth in body height. *The Angle Orthodontist*.

[6] Proffit W. R., Fields H. W., Moray L. J. (1998). Prevalence of malocclusion and orthodontic treatment need in the United States: estimates from the NHANES III survey. *The International Journal of Adult Orthodontics and Orthognathic Surgery*.

[7] Perinetti G., Cordella C., Pellegrini F., Esposito P. (2008). The prevalence of malocclusal traits and their correlations in mixed dentition children: results from the Italian OHSAR Survey. *Oral Health and Preventive Dentistry*.

[8] McNamara J. A., Bookstein F. L., Shaughnessy T. G. (1985). Skeletal and dental changes following functional regulator therapy on class II patients. *American Journal of Orthodontics*.

[9] McNamara J. A., Hinton R. J., Hoffman D. L. (1982). Histologic analysis of temporomandibular joint adaptation to protrusive function in young adult rhesus monkeys (Macaca mulatta). *American Journal of Orthodontics*.

[10] Proff P., Gedrange T., Franke R. (2007). Histological and histomorphometric investigation of the condylar cartilage of juvenile pigs after anterior mandibular displacement. *Annals of Anatomy*.

[11] Marsico E., Gatto E., Burrascano M., Matarese G., Cordasco G. (2011). Effectiveness of orthodontic treatment with functional appliances on mandibular growth in the short term. *American Journal of Orthodontics and Dentofacial Orthopedics*.

[12] Chen J. Y., Will L. A., Niederman R. (2002). Analysis of efficacy of functional appliances on mandibular growth. *American Journal of Orthodontics and Dentofacial Orthopedics*.

[13] Thiruvenkatachari B., Harrison J. E., Worthington H. V., O'Brien K. D. (2013). Orthodontic treatment for prominent upper front teeth (Class II malocclusion) in children. *Cochrane Database of Systematic Reviews*.

[14] Ehsani S., Nebbe B., Normando D., Lagravere M. O., Flores-Mir C. (2014). Short-term treatment effects produced by the Twin-block appliance: a systematic review and meta-analysis. *The European Journal of Orthodontics*.

[15] Koretsi V., Zymperdikas V. F., Papageorgiou S. N., Papadopoulos M. A. (2015). Treatment effects of removable functional appliances in patients with Class II malocclusion: a systematic review and meta-analysis. *European Journal of Orthodontics*.

[16] Zymperdikas V. F., Koretsi V., Papageorgiou S. N., Papadopoulos M. A. (2016). Treatment effects of fixed functional appliances in patients with Class II malocclusion: a systematic review and meta-analysis. *European Journal of Orthodontics*.

[17] Perinetti G., Primožič J., Furlani G., Franchi L., Contardo L. (2015). Treatment effects of fixed functional appliances alone or in combination with multibracket appliances: a systematic review and meta-analysis. *The Angle Orthodontist*.

[18] Perinetti G., Primožič J., Franchi L., Contardo L. (2015). Treatment effects of removable functional appliances in pre-pubertal and pubertal Class II patients: a systematic review and meta-analysis of controlled studies. *PLoS ONE*.

[19] Antonarakis G. S., Kiliaridis S. (2007). Short-term anteroposterior treatment effects of functional appliances and extraoral traction on class II malocclusion. A meta-analysis. *The Angle Orthodontist*.

[20] Baccetti T., Franchi L., McNamara J. A. (2005). The cervical vertebral maturation (CVM) method for the assessment of optimal treatment timing in dentofacial orthopedics. *Seminars in Orthodontics*.

[21] Perinetti G., Contardo L., Castaldo A., McNamara J. A., Franchi L. (2016). Diagnostic reliability of the cervical vertebral maturation method and standing height in the identification of the mandibular growth spurt. *The Angle Orthodontist*.

[22] Franchi L., Alvetro L., Giuntini V., Masucci C., Defraia E., Baccetti T. (2011). Effectiveness of comprehensive fixed appliance treatment used with the Forsus Fatigue Resistant Device in Class II patients. *The Angle Orthodontist*.

[23] Baccetti T., Franchi L., Stahl F. (2009). Comparison of 2 comprehensive Class II treatment protocols including the bonded Herbst and headgear appliances: a double-blind study of consecutively treated patients at puberty. *American Journal of Orthodontics & Dentofacial Orthopedics*.

[24] Martina R., Cioffi I., Galeotti A. (2013). Efficacy of the Sander bite-jumping appliance in growing patients with mandibular retrusion: a randomized controlled trial. *Orthodontics and Craniofacial Research*.

[25] Baccetti T., Franchi L., Toth L. R., McNamara J. A. (2000). Treatment timing for Twin-block therapy. *American Journal of Orthodontics and Dentofacial Orthopedics*.

[26] Nalbantgil D., Arun T., Sayinsu K., Işik F. (2005). Skeletal, dental and soft-tissue changes induced by the Jasper Jumper appliance in late adolescence. *The Angle Orthodontist*.

[27] Tulloch J. F., Phillips C., Koch G., Proffit W. R. (1997). The effect of early intervention on skeletal pattern in Class II malocclusion: a randomized clinical trial. *American Journal of Orthodontics and Dentofacial Orthopedics*.

[28] Perillo L., Femiano A., Palumbo S., Contardo L., Perinetti G. (2013). Skeletal and dental effects produced by functional regulator-2 in pre-pubertal class II patients: a controlled study. *Progress in Orthodontics*.

[29] O'Brien K., Wright J., Conboy F. (2003). Effectiveness of early orthodontic treatment with the Twin-block appliance: a multicenter, randomized, controlled trial. Part 1: dental and skeletal effects. *American Journal of Orthodontics & Dentofacial Orthopedics*.

[30] Greulich W. W., Pyle S. I. (1959). *Radiographic Atlas of Skeletal Development of the Hand and Wrist*.

[31] Chertkow S. (1980). Tooth mineralization as an indicator of the pubertal growth spurt. *American Journal of Orthodontics*.

[32] Hägg U., Taranger J. (1982). Maturation indicators and the pubertal growth spurt. *American Journal of Orthodontics*.

[33] Hassel B., Farman A. G. (1995). Skeletal maturation evaluation using cervical vertebrae. *American Journal of Orthodontics & Dentofacial Orthopedics*.

[34] Grave K. C. (1973). Timing of facial growth: a study of relations with stature and ossification in the hand around puberty. *Australian Orthodontic Journal*.

[35] Fishman L. S. (1982). Radiographic evaluation of skeletal maturation. A clinically oriented method based on hand-wirst films. *The Angle Orthodontist*.

[36] Rajagopal R., Kansal S. (2002). A comparison of modified MP3 stages and the cervical vertebrae as growth indicators. *Journal of Clinical Orthodontics*.

[37] Perinetti G., Sbardella V., Contardo L. (2016). Diagnostic reliability of the third finger middle phalanx maturation (MPM) method in the identification of the mandibular growth peak. *The European Journal of Orthodontics*.

[38] Abdel-Kader H. M. (1998). The reliability of dental x-ray film in assessment of MP3 stages of the pubertal growth spurt. *American Journal of Orthodontics and Dentofacial Orthopedics*.

[39] Lamparski D. G. (1972). *Skeletal Age Assessment Utilizing Cervical Vertebrae*.

[40] Demirjian A., Goldstein H., Tanner J. M. (1973). A new system of dental age assessment. *Human Biology*.

[41] Perinetti G., Baccetti T., Di Leonardo B., Di Lenarda R., Contardo L. (2011). Dentition phase and chronological age in relation to gingival crevicular fluid alkaline phosphatase activity in growing subjects. *Progress in Orthodontics*.

[42] Masoud M., Masoud I., Kent R. L., Gowharji N., Cohen L. E. (2008). Assessing skeletal maturity by using blood spot insulin-like growth factor I (IGF-I) testing. *American Journal of Orthodontics and Dentofacial Orthopedics*.

[43] Ishaq R. A. R., Soliman S. A. Z., Foda M. Y., Fayed M. M. S. (2012). Insulin-like growth factor I: a biologic maturation indicator. *American Journal of Orthodontics and Dentofacial Orthopedics*.

[44] Perinetti G., Baccetti T., Contardo L., Di Lenarda R. (2011). Gingival crevicular fluid alkaline phosphatase activity as a non-invasive biomarker of skeletal maturation. *Orthodontics and Craniofacial Research*.

[45] Perinetti G., Franchi L., Castaldo A., Contardo L. (2012). Gingival crevicular fluid protein content and alkaline phosphatase activity in relation to pubertal growth phase. *The Angle Orthodontist*.

[46] Baccetti T., Franchi L., McNamara J. A. (2002). An improved version of the cervical vertebral maturation (CVM) method for the assessment of mandibular growth. *The Angle Orthodontist*.

[47] Ball G., Woodside D., Tompson B., Hunter W. S., Posluns J. (2011). Relationship between cervical vertebral maturation and mandibular growth. *American Journal of Orthodontics and Dentofacial Orthopedics*.

[48] Beit P., Peltomäki T., Schätzle M., Signorelli L., Patcas R. (2013). Evaluating the agreement of skeletal age assessment based on hand-wrist and cervical vertebrae radiography. *American Journal of Orthodontics and Dentofacial Orthopedics*.

[49] Franchi L., Baccetti T., McNamara J. A. (2000). Mandibular growth as related to cervical vertebral maturation and body height. *American Journal of Orthodontics and Dentofacial Orthopedics*.

[50] Fudalej P., Bollen A.-M. (2010). Effectiveness of the cervical vertebral maturation method to predict postpeak circumpubertal growth of craniofacial structures. *American Journal of Orthodontics and Dentofacial Orthopedics*.

[51] Grave K., Townsend G. (2003). Cervical vertebral maturation as a predictor of the adolescent growth spurt. *Australian Orthodontic Journal*.

[52] Gu Y., McNamara J. A. (2007). Mandibular growth changes and cervical vertebral maturation: a cephalometric implant study. *The Angle Orthodontist*.

[53] Mellion Z. J., Behrents R. G., Johnston L. E. (2013). The pattern of facial skeletal growth and its relationship to various common indexes of maturation. *American Journal of Orthodontics and Dentofacial Orthopedics*.

[54] Houston W. J. B. (1980). Relationships between skeletal maturity estimated from hand-wrist radiographs and the timing of the adolescent growth spurt. *European Journal of Orthodontics*.

[55] Houston W. J., Miller J. C., Tanner J. M. (1979). Prediction of the timing of the adolescent growth spurt from ossification events in hand-wrist films. *British Journal of Orthodontics*.

[56] Altan M., Nebioğlu Dalcı Ö., Işeri H. (2012). Growth of the cervical vertebrae in girls from 8 to 17 years. A longitudinal study. *European Journal of Orthodontics*.

[57] Perinetti G., Contardo L., Baccetti T., McNamara J. A., Hatch N., Kapila S. D. (2012). Gingival crevicular fluid as a source of biomarkers of patient responsiveness to orthodontic treatment. *Taking Advantage of Emerging Technologies in Clinical Practice*.

[58] Flores-Mir C., Burgess C. A., Champney M., Jensen R. J., Pitcher M. R., Major P. W. (2006). Correlation of skeletal maturation stages determined by cervical vertebrae and hand-wrist evaluations. *The Angle Orthodontist*.

[59] Wong R. W. K., Alkhal H. A., Rabie A. B. M. (2009). Use of cervical vertebral maturation to determine skeletal age. *American Journal of Orthodontics and Dentofacial Orthopedics*.

[60] Różyło-Kalinowska I., Kolasa-Rączka A., Kalinowski P. (2011). Relationship between dental age according to Demirjian and cervical vertebrae maturity in Polish children. *European Journal of Orthodontics*.

[61] Gray S., Bennani H., Kieser J. A., Farella M. (2016). Morphometric analysis of cervical vertebrae in relation to mandibular growth. *American Journal of Orthodontics and Dentofacial Orthopedics*.

[62] Engel T. P., Renkema A.-M., Katsaros C., Pazera P., Pandis N., Fudalej P. S. (2015). The cervical vertebrae maturation (CVM) method cannot predict craniofacial growth in girls with Class II malocclusion. *European Journal of Orthodontics*.

[63] Bishara S. E., Jamison J. E., Peterson L. C., DeKock W. H. (1981). Longitudinal changes in standing height and mandibular parameters between the ages of 8 and 17 years. *American Journal of Orthodontics*.

[64] O'Reilly M. T., Yanniello G. J. (1988). Mandibular growth changes and maturation of cervical vertebrae. A longitudinal cephalometric study. *The Angle Orthodontist*.

[65] Tofani M. I. (1972). Mandibular growth at puberty. *American Journal of Orthodontics*.

[66] Soegiharto B. M., Moles D. R., Cunningham S. J. (2008). Discriminatory ability of the skeletal maturation index and the cervical vertebrae maturation index in detecting peak pubertal growth in Indonesian and white subjects with receiver operating characteristics analysis. *American Journal of Orthodontics and Dentofacial Orthopedics*.

[67] Oztoprak M. O., Nalbantgil D., Uyanlar A., Arun T. (2012). A cephalometric comparative study of class II correction with Sabbagh universal spring (SUS^2^) and forsus FRD appliances. *European Journal of Dentistry*.

[68] Küçükkeleş N., Sandalli T. (1992). Cephalometric evaluation of the therapeutic effects of the Herbst appliance in the treatment of Class II. Div I. malocclusion. *Journal of Marmara University Dental Faculty*.

[69] Björk A. (1947). The face in profile. *Svensk Tandlarkare Tidskrift*.

[70] Haas D. W., Martinez F., Eckert G. J., Diers N. R. (2001). Measurements of mandibular length: a comparison of articulare vs condylion. *The Angle Orthodontist*.

[71] Stickel A., Pancherz H. (1988). Can ‘articulare’ be used in the cephalometric analysis of mandibular length? A methodologic study. *European Journal of Orthodontics*.

[72] Aelbers C. M., Dermaut L. R. (1996). Orthopedics in orthodontics: part I, fiction or reality—a review of the literature. *American Journal of Orthodontics and Dentofacial Orthopedics*.

[73] Todd T. W. (1937). *Atlas of Skeletal Maturation*.

[74] Sutow W. W., Ohwada W. (1953). Skeletal maturation in healthy Japanese children from 6 to 19 years of age. Comparison with skeletal maturation in American children. *Hiroshima Journal of Medical Sciences*.

[75] Tanner J. M., Whitehouse R. W. (1982). *Atlas of Children's Growth: Normal Variation and Growth Disorders*.

[76] Grave K. C., Brown T. (1976). Skeletal ossification and the adolescent growth spurt. *American Journal of Orthodontics*.

[77] Leite H. R., O'Reilly M. T., Close J. M. (1987). Skeletal age assessment using the first, second, and third fingers of the hand. *American Journal of Orthodontics and Dentofacial Orthopedics*.

[78] Perinetti G., Perillo L., Franchi L., Di Lenarda R., Contardo L. (2014). Maturation of the middle phalanx of the third finger and cervical vertebrae: a comparative and diagnostic agreement study. *Orthodontics and Craniofacial Research*.

[79] Donatelli R. E., Lee S.-J. (2013). How to report reliability in orthodontic research: part 1. *American Journal of Orthodontics and Dentofacial Orthopedics*.

[80] Flores-Mir C., Nebbe B., Major P. W. (2004). Use of skeletal maturation based on hand-wrist radiographic analysis as a predictor of facial growth: a systematic review. *Angle Orthodontist*.

[81] Karlberg J. (2002). Secular trends in pubertal development. *Hormone Research*.

[82] Parent A.-S., Teilmann G., Juul A., Skakkebaek N. E., Toppari J., Bourguignon J.-P. (2003). The timing of normal puberty and the age limits of sexual precocity: variations around the world, secular trends, and changes after migration. *Endocrine Reviews*.

[83] Silveira A. M., Fishman L. S., Subtelny J. D., Kassebaum D. K. (1992). Facial growth during adolescence in early, average and late maturers. *The Angle Orthodontist*.

[84] Mitani H., Sato K. (1992). Comparison of mandibular growth with other variables during puberty. *The Angle Orthodontist*.

[85] Helm S., Siersbaek-Nielsen S., Skieller V., Björk A. (1971). Skeletal maturation of the hand in relation to maximum puberal growth in body height. *Tandlaegebladet*.

[86] Navlani M., Makhija P. G. (2013). Evaluation of skeletal and dental maturity indicators and assessment of cervical vertebral maturation stages by height/width ratio of third cervical vertebra. *Journal of Pierre Fauchard Academy (India Section)*.

[87] Özer T., Kama J. D., Özer S. Y. (2006). A practical method for determining pubertal growth spurt. *American Journal of Orthodontics and Dentofacial Orthopedics*.

[88] Prasad M., Ganji V. S. K., George S. A., Talapaneni A. K., Shetty S. K. (2013). A comparison between cervical vertebrae and modified MP3 stages for the assessment of skeletal maturity. *Journal of Natural Science, Biology and Medicine*.

[89] Kuc-Michalska M., Baccetti T. (2010). Duration of the pubertal peak in skeletal class I and Class III subjects. *The Angle Orthodontist*.

[90] Salazar-Lazo R., Arriola-Guillen L. E., Flores-Mir C. (2014). Duration of the peak of adolescent growth spurt in class I and II malocclusion subjects using a cervical vertebrae maturation analisis. *Acta Odontológica Latinoamericana*.

[91] San Román P., Palma J. C., Oteo M. D., Nevado E. (2002). Skeletal maturation determined by cervical vertebrae development. *European Journal of Orthodontics*.

[92] Uysal T., Ramoglu S. I., Basciftci F. A., Sari Z. (2006). Chronologic age and skeletal maturation of the cervical vertebrae and hand-wrist: is there a relationship?. *American Journal of Orthodontics and Dentofacial Orthopedics*.

[93] Perinetti G., Primozic J., Franchi L., Contardo L. (2016). Cervical vertebral maturation method: growth timing versus growth amount. *European Journal of Orthodontics*.

[94] Chen L.-L., Xu T.-M., Jiang J.-H., Zhang X.-Z., Lin J.-X. (2008). Quantitative cervical vertebral maturation assessment in adolescents with normal occlusion: a mixed longitudinal study. *American Journal of Orthodontics and Dentofacial Orthopedics*.

[95] Perinetti G. (2014). Cervical vertebral maturation: is that all?. *American Journal of Orthodontics and Dentofacial Orthopedics*.

[96] Gabriel D. B., Southard K. A., Qian F., Marshall S. D., Franciscus R. G., Southard T. E. (2009). Cervical vertebrae maturation method: poor reproducibility. *American Journal of Orthodontics and Dentofacial Orthopedics*.

[97] Nestman T. S., Marshall S. D., Qian F., Holton N., Franciscus R. G., Southard T. E. (2011). Cervical vertebrae maturation method morphologic criteria: poor reproducibility. *American Journal of Orthodontics and Dentofacial Orthopedics*.

[98] Perinetti G., Caprioglio A., Contardo L. (2014). Visual assessment of the cervical vertebral maturation stages a study of diagnostic accuracy and repeatability. *The Angle Orthodontist*.

[99] Patcas R., Signorelli L., Peltomäki T., Schätzle M. (2012). Is the use of the cervical vertebrae maturation method justified to determine skeletal age? A comparison of radiation dose of two strategies for skeletal age estimation. *European Journal of Orthodontics*.

[100] Tassi N. G. G., Franchi L., Baccetti T., Barbato E. (2007). Diagnostic performance study on the relationship between the exfoliation of the deciduous second molars and the pubertal growth spurt. *American Journal of Orthodontics and Dentofacial Orthopedics*.

[101] Franchi L., Baccetti T., De Toffol L., Polimeni A., Cozza P. (2008). Phases of the dentition for the assessment of skeletal maturity: a diagnostic performance study. *American Journal of Orthodontics and Dentofacial Orthopedics*.

[102] Gianelly A. A. (1995). One-phase versus two-phase treatment. *American Journal of Orthodontics and Dentofacial Orthopedics*.

[103] Wieslander L. (1975). Early or late cervical traction therapy of Class II malocclusion in the mixed dentition. *American Journal of Orthodontics*.

[104] Perinetti G., Di Lenarda R., Contardo L. (2013). Diagnostic performance of combined canine and second molar maturity for identification of growth phase. *Progress in Orthodontics*.

[105] Perinetti G., Contardo L., Gabrieli P., Baccetti T., Di Lenarda R. (2012). Diagnostic performance of dental maturity for identification of skeletal maturation phase. *European Journal of Orthodontics*.

[106] Coutinho S., Buschang P. H., Miranda F. (1993). Relationships between mandibular canine calcification stages and skeletal maturity. *American Journal of Orthodontics and Dentofacial Orthopedics*.

[107] Krailassiri S., Anuwongnukroh N., Dechkunakorn S. (2002). Relationships between dental calcification stages and skeletal maturity indicators in Thai individuals. *The Angle Orthodontist*.

[108] Chen J., Hu H., Guo J. (2010). Correlation between dental maturity and cervical vertebral maturity. *Oral Surgery, Oral Medicine, Oral Pathology, Oral Radiology, and Endodontics*.

[109] Kumar S., Singla A., Sharma R., Virdi M. S., Anupam A., Mittal B. (2012). Skeletal maturation evaluation using mandibular second molar calcification stages. *The Angle Orthodontist*.

[110] Cericato G. O., Franco A., Bittencourt M. A. V., Nunes M. A. P., Paranhos L. R. (2016). Correlating skeletal and dental developmental stages using radiographic parameters. *Journal of Forensic and Legal Medicine*.

[111] Uysal T., Sari Z., Ramoglu S. I., Basciftci F. A. (2004). Relationships between dental and skeletal maturity in Turkish subjects. *The Angle Orthodontist*.

[112] Surendran S., Thomas E. (2014). Tooth mineralization stages as a diagnostic tool for assessment of skeletal maturity. *American Journal of Orthodontics and Dentofacial Orthopedics*.

[113] Sukhia R. H., Fida M. (2010). Correlation among chronologic age, skeletal maturity, and dental age. *World Journal of Orthodontics*.

[114] Sierra A. M. (1987). Assessment of dental and skeletal maturity. A new approach. *The Angle Orthodontist*.

[115] Lopes L. J., De Oliveira Gamba T., Visconti M. A. P. G., Ambrosano G. M. B., Haiter-Neto F., Freitas D. Q. (2016). Utility of panoramic radiography for identification of the pubertal growth period. *American Journal of Orthodontics and Dentofacial Orthopedics*.

[116] Başaran G., Özer T., Hamamci N. (2007). Cervical vertebral and dental maturity in Turkish subjects. *American Journal of Orthodontics and Dentofacial Orthopedics*.

[117] Bagherpour A., Pousti M., Adelianfar E. (2014). Hand skeletal maturity and its correlation with mandibular dental development. *Journal of Clinical and Experimental Dentistry*.

[118] Perinetti G., Westphalen G. H., Biasotto M., Salgarello S., Contardo L. (2013). The diagnostic performance of dental maturity for identification of the circumpubertal growth phases: a meta-analysis. *Progress in Orthodontics*.

[119] Bambha J. K. (1961). Longitudinal cephalometric roentgenographic study of face and cranium in relation to body height. *Journal of the American Dental Association*.

[120] Hunter C. J. (1966). The correlation of facial growth with body height and skeletal maturation at adolescence. *The Angle Orthodontist*.

[121] Bergersen E. O. (1972). The male adolescent facial growth spurt: its prediction and relation to skeletal maturation. *The Angle Orthodontist*.

[122] Bowden B. D. (1976). Epiphysial changes in the hand/wrist area as indicators of adolescent stage. *Australian Orthodontic Journal*.

[123] Tanner J. M., Whitehouse R. H., Marubini E., Resele L. F. (1976). The adolescent growth spurt of boys and girls of the Harpenden growth study. *Annals of Human Biology*.

[124] Baccetti T., Franchi L., De Toffol L., Ghiozzi B., Cozza P. (2006). The diagnostic performance of chronologic age in the assessment of skeletal maturity. *Progress in Orthodontics*.

[125] Hägg U., Taranger J. (1980). Menarche and voice change as indicators of the pubertal growth spurt. *Acta Odontologica Scandinavica*.

[126] Lai E. H.-H., Chang J. Z.-C., Jane Yao C.-C. (2008). Relationship between age at menarche and skeletal maturation stages in Taiwanese female orthodontic patients. *Journal of the Formosan Medical Association*.

[127] Masoud M. I., Marghalani H. Y. A., Masoud I. M., Gowharji N. F. (2012). Prospective longitudinal evaluation of the relationship between changes in mandibular length and blood-spot IGF-1 measurements. *American Journal of Orthodontics and Dentofacial Orthopedics*.

[128] Perinetti G., Contardo L. (2016). Gingival crevicular fluid alkaline phosphatase activity in relation to pubertal growth spurt and dental maturation: a multiple regression study. *South European Journal of Orthodontics and Dentofacial Research*.

[129] Perinetti G., Di Leonardo B., Di Lenarda R., Contardo L. (2013). Repeatability of gingival crevicular fluid collection and quantification, as determined through its alkaline phosphatase activity: implications for diagnostic use. *Journal of Periodontal Research*.

[130] Perinetti G., Paolantonio M., Femminella B., Serra E., Spoto G. (2008). Gingival crevicular fluid alkaline phosphatase activity reflects periodontal healing/recurrent inflammation phases in chronic periodontitis patients. *Journal of Periodontology*.

[131] Al-Jewair T. S. (2015). Meta-analysis on the mandibular dimensions effects of the MARA appliance in patients with Class II malocclusions. *The Angle Orthodontist*.

[132] Perillo L., Cannavale R., Ferro F. (2011). Meta-analysis of skeletal mandibular changes during Fränkel appliance treatment. *European Journal of Orthodontics*.

[133] Yang X., Zhu Y., Long H. (2016). The effectiveness of the Herbst appliance for patients with Class II malocclusion: a meta-analysis. *European Journal of Orthodontics*.

[134] Cozza P., Baccetti T., Franchi L., De Toffol L., McNamara J. A. (2006). Mandibular changes produced by functional appliances in Class II malocclusion: a systematic review. *American Journal of Orthodontics and Dentofacial Orthopedics*.

[135] Franchi L., Baccetti T. (2006). Prediction of individual mandibular changes induced by functional jaw orthopedics followed by fixed appliances in Class II patients. *The Angle Orthodontist*.

[136] Wheeler T. T., McGorray S. P., Dolce C., Taylor M. G., King G. J. (2002). Effectiveness of early treatment of Class II malocclusion. *American Journal of Orthodontics and Dentofacial Orthopedics*.

[137] Flores-Mir C. (2007). Can we extract useful and scientifically sound information from retrospective nonrandomized trials to be applied in orthodontic evidence-based practice treatments?. *American Journal of Orthodontics and Dentofacial Orthopedics*.

[138] Shrier I., Boivin J.-F., Steele R. J. (2007). Should meta-analyses of interventions include observational studies in addition to randomized controlled trials? A critical examination of underlying principles. *American Journal of Epidemiology*.

[139] Darendeliler M. A. (2006). Validity of randomized clinical trials in evaluating the outcome of Class II treatment. *Seminars in Orthodontics*.

[140] de Almeida-Pedrin R. R., Rodrigues de Almeida M., Rodrigues de Almeida R., Pinzan A., Ferreira F. P. C. (2007). Treatment effects of headgear biteplane and bionator appliances. *American Journal of Orthodontics and Dentofacial Orthopedics*.

[141] Quintão C., Helena I., Brunharo V. P., Menezes R. C., Almeida M. A. O. (2006). Soft tissue facial profile changes following functional appliance therapy. *European Journal of Orthodontics*.

[142] Pancherz H. (1982). The mechanism of Class II correction in Herbst appliance treatment. A cephalometric investigation. *American Journal of Orthodontics*.

[143] Mathews J. R., Ware W. H. (1978). Longitudinal mandibular growth in children with tantalum implants. *American Journal of Orthodontics*.

